# Maternal education level and maternal healthcare utilization in the Democratic Republic of the Congo: an analysis of the multiple indicator cluster survey 2017/18

**DOI:** 10.1186/s12913-021-06854-x

**Published:** 2021-08-21

**Authors:** Hanyu Wang, Eric Frasco, Rie Takesue, Kun Tang

**Affiliations:** 1 Vanke School of Public Health, Tsinghua University, 30 Shuangqing Rd, Haidian District, 100084 Beijing, China; 2 Institute for Global Health, University College London, Gower St, Bloomsbury, WC1E 6BT London, UK; 3 Health Section Programme Division, UNICEF Headquarters, 3 United Nations Plaza, NY 10017 New York, USA

**Keywords:** Maternal Education, Maternal Health Utilization, The Democratic Republic of the Congo, Social Determinants of Health

## Abstract

**Background:**

Understanding how socioeconomic factors influence maternal health services utilization is crucial to reducing preventable maternal deaths in the DRC. Maternal education is considered an important associate of maternal health service utilization. This study aims to investigate the association between maternal education and the utilization of maternal health services, as well as present geographical and socio-economic disparities in the utilization.

**Methods:**

The MICS survey was employed as the data source, which is a nationally representative survey conducted from 2017 to 2018 in the DRC. The exposure for this study was the maternal education level, which was categorized into three groups: (1) below primary and none, (2) primary and (3) secondary and above. Prenatal care indicators included: if the mother ever received prenatal care, if the mother had antenatal checks no less than four times, and if a skilled attendant was present at birth. Postnatal care indicators included: if the mother received postnatal care and if the baby was checked after birth. Emergency obstetric interventions were indicted by cesarean sections. Descriptive analyses and logistic regressions were used as analytical methods.

**Results:**

Of all 8,560 participants included, 21.88 % had below primary school or no education, 39.81 % had primary school education, and 38.31 % had secondary education or above. The majority of participants were from rural areas, except for Kinshasa. Overall, a better education was associated with higher utilization of antenatal care. A dose-response effect was also observed. Compared to women with below primary or no education, women with secondary and above education were more likely to receive cesarean sections. Wealth status, as well as rural and urban division, modified the associations.

**Conclusions:**

Mothers’ education level is an important associate for utilizing appropriate maternal healthcare, with wealth and region as modifying factors. Educational levels should be considered when designing public health interventions and women’s empowerment programs in the DRC. For example, relevant programs need to stratify the interventions according to educational attainment.

**Supplementary Information:**

The online version contains supplementary material available at 10.1186/s12913-021-06854-x.

## Background

The World Health Organization (WHO) estimated that every day in 2017, there was an average of 810 maternal deaths from preventable causes worldwide. Ninety-four per cent of these deaths occurred in low and lower-middle-income countries, with one-third in sub-Saharan Africa [1]. The WHO also stressed inadequate and poor maternal health services and inequality in access as key factors in sub-Saharan Africa’s high maternal mortality ratio [2]. In sub-Saharan Africa, although significant progress has been made in recent decades, young pregnant women are still facing enormous challenges in accessing and utilizing adequate maternal health services. This includes prenatal care, postnatal care, and emergency obstetric interventions [2, 3].

The Democratic Republic of the Congo (DRC) is the largest country in sub-Saharan Africa with a modern history marked by violent conflict, political turmoil, resource exploitation, and communicable disease epidemics. A large proportion of its population is living in poverty due to resource plundering and governance problems [4]. Furthermore, a 2015 estimate by the United Nations Maternal Mortality Estimation Inter-Agency Group indicated that the maternal mortality ratio in the DRC was estimated at 693 per 100,000 live births in 2015, making it one of the highest in the world[5] The report noted that the estimate could subject to underestimation[6], as the country’s paper-based health information system could impact the timeliness of the report of maternal mortality. The Demographic and Health Surveys in the DRC indicated that maternal health service utilization, including antenatal care, postnatal care, and having skilled attendants at birth, was relatively poor in the country and disparities were prevalent across geographical locations and socioeconomic status [5, 7].

Socioeconomic status at an individual level has been associated with maternal care coverage in both developed [8] and developing countries [9]. Those with higher socioeconomic status, including more wealth and better education, are generally considered to have higher access to maternal health services in developing countries [8–10]. Education level is an important and unique social determinant of maternal health service utilization. It is one of the key indicators of socioeconomic status and is known to positively impact health service utilization [11]. Meanwhile, higher education status generally brings better literary and better health-seeking behaviors [11]. Education is also considered an essential means to empower women [12]. Therefore, exploring and understanding the association between maternal education and utilization of maternal health services not only has implications for public health but has the potential to contribute to gender equality on the sub-Saharan continent.

Nevertheless, there are a limited number of studies conducted in the DRC to assess the association between maternal education and maternal health service utilization. A study conducted in Lubumbashi city in the DRC observed that the overall coverage of maternal health services was low, with parity and reasons for pregnancy being associated with the utilization of maternal health services [13]. One recent study that assesses the determinants of antenatal care in the DRC in endemic malaria settings found that educational level was significantly associated with the first visit to the antenatal care facilities. The study also observed that marital status and geographical locations are related to the utilization of antennal care services [14]. The study, however, focused on the western part of the country with high malaria prevalence and therefore could not draw policy implications at the national level. Another study using the Demographic and Health Survey in 2007 found that facility-based delivery is associated with both the mother and her partners’ education [15]. The study, however, used data that were collected more than 10 years ago and might therefore undermine their relevance today.

To date, there are very few, if any, studies to explore maternal health service utilization and its association with maternal education in the DRC, with population-based research particularly lacking. Using a large population-based database provided by UNICEF, the present study aims to investigate the association between maternal education and maternal health services utilization, including prenatal care, provision of antenatal checks at least four times before birth, postnatal care, and cesarean section in the DRC, with considerations of rural-urban and economic disparity. Additionally, this study presents the geographical disparity of access to maternal health services by administrative regions in the DRC to further guide policymaking.

## Methods

### Data Source

We employed the MICS, conducted from 2017 to 2018 in the DRC, as the data source. This is the fourth MICS conducted in the DRC. MICS is one of the largest household survey programs in the world and has covered 116 countries since it was initiated 25 years ago. Details about the MICS study can be found on the official website [16].

This survey was conducted in the DRC by the National Bureau of Statistics, with support from UNICEF and USAID/PMI from 2017 to 2018. The sampling methods guaranteed a nationally representative sample. Multi-stage stratified cluster sampling was employed for the selection of the survey sample to generate a nationally representative database. Both urban and rural areas across all 26 provinces in the DRC were included in the sample design to generate a representative sample. Urban and rural areas were identified as the main sampling strata using a two-stage selection method. A detailed description of the sampling mechanism and the survey design can be found in the MICS-Palu 2018 report (in French) [17]. This survey generates more than 150 key indicators, providing disaggregated data on the situation of children and women and the living conditions of populations in the areas of health, nutrition, education, protection, hygiene, and sanitation. The original questionnaire was in French.

In the current analysis, women ages 15–49 were included, with an original sample size of 21,756 (women who were interviewed). This study focused on questions related to maternal health, with many questions designed only for women who have given birth in the past two years. As the questions were based on mothers’ memory of details from their most recent birth, including only women who have given birth in the last two years can also reduce recall biases. Accordingly, we included those women who have given birth in the last two years (final sample size, *N* = 8,560).

### Studied variables

#### Exposures

The exposure in this study is the maternal education level. The original question from the survey was: “Niveau d’instruction de la femme (Women’s educational level).” A categorical variable was generated from this question that included five categories: pre-primary and below, primary, secondary level 1, secondary level 2, and above secondary. To balance the sample size in each category and draw meaningful comparisons between the categories, maternal education level was categorized into three groups: below primary and none, primary, and secondary and above.

#### Outcomes

We reviewed the database and the questionnaire and employed six main indicators for maternal health services utilization to present the situation concerning prenatal care, postnatal care, and emergency obstetric interventions. Prenatal care indicators included if the mother ever received prenatal care if the mother had antenatal checks no less than four times. times, Skilled attendant was present at birth was also included as an indicator of maternity care services. Ever received prenatal care was defined as 1 if mothers answered yes to the question: “*Have you been to a prenatal consultation during pregnancy?*” Antenatal checks no less than four times (ANC ≥ 4) were defined as 1 if mothers filled in a number that was more than four to the question “*How many times did you visit prenatal consultation during this pregnancy?*” and was seen by a health professional (doctor, nurse, or midwife) during the antenatal checks. Skilled attendants at birth included doctors, nurses, and midwives. These three variables were derived directly from relevant categorical questions in the survey. Postnatal care indicators included if the mother received postnatal care (PNC) and if the baby was checked after birth. Mother received PNC was defined as the mother received postnatal health check within two days after delivery, which was indicated by the question: “*After birth, was your health checked?*” and “*How long after delivery did this check take place?*”. Baby received PNC was defined similarly as the baby received postnatal health check within two days after birth. Emergency obstetric interventions were indicted by whether the mother received cesarean sections.

#### Other co-variates

Socioeconomic characteristics apart from maternal education were collected from the MICS survey. In this study, we considered a range of socioeconomic variables that might confound or modify the association between maternal education and maternal health services utilization. Maternal age was collected and used in the analysis as a continuous variable. Urban and rural divisions were collected based on the households’ residency. Provinces were based on participants’ residential regions and were classified into twenty-six administrative regions based on the DRC official classification. Wealth was accessed in the survey as a categorical variable and presented as quantiles in the survey. We further defined “low-income” as wealth below the fortieth percentile and “middle-high-income” as wealth above the fortieth percentile. Marital status was collected as “currently married/in union”, “formerly married/in union”, and “never married/in union”. Age at first marriage was collected and adjusted in the model as a continuous variable. We further classified it as “first marriage < 18” and “first marriage ≥ 18” for stratification purposes. Participants’ health behaviors, including smoking status and alcohol use, were assessed by self-reported lifestyle questions and classified as smoker/drinker and non-smoker/drinker. Participants’ disability status was also accessed in the survey by asking “if you have functional disability” and was collected as a binary variable.

### Data analysis

Descriptive analyses were used to illustrate the basic socioeconomic and maternal health services utilization situation in twenty-six administrative regions. Relevant maps were drawn using the “spmap” function in Stata 14 [18]. The association between maternal education and maternal health services utilization indicators was analyzed using logistic regression models. In the analysis, survey design and sample weights were taken into consideration by adding weights when tabulating the participants’ characteristics and computing the odds ratios (ORs). Sample design and weight considerations were achieved by using “svyset” and “svy” functions in Stata [19]. Weight and strata used in the analysis were included in the MICS database. All the ORs were presented with 95 % confidence intervals (CIs). The below primary and non-educated group was chosen to be a reference group in all of the models. Two models were fitted: (1) unadjusted; (2) adjusted for maternal age, urban/rural, province (26 administrative regions), wealth quantile, marital status, age at first marriage, tobacco/alcohol use, and disability. The adjusted variables were chosen by reviewing relevant literature. To further understand the relationship and the modifying effects of other socioeconomic variables, we stratified the analysis by rural/urban, low income and middle-high-income, and age at first marriage (< 18 and ≥ 18). All analyses were conducted by Stata 14 (StataCorp).

## Results

### Characteristics and the geographic distributions of the studied variables

Table 1 presents the characteristics of the study population by maternal education. Of all 8,560 participants included, 21.88 % had below primary school or no education, 39.81 % primary school education, and 38.31 % had secondary and above education. The mean maternal ages were 30.22 (SD = 0.33), 29.30 (SD = 0.26), and 27.54 (SD = 0.19) for the three maternal education groups respectively. Those who had a secondary and above education were more likely to live in urban areas (58.56 %), compared to those who had below primary and no education (14.60 %) and those who had primary school education (22.63 %). The population with secondary and above education also had a higher percentage of participants in the highest income percentile (56.24 %). Women with higher education were more likely to consume alcohol. The distributions of marital status, age at first marriage, and smoking status were similar across all maternal education groups. Insurance coverage was higher for women with secondary and above education (6.93 %), compared to two other groups. For utilization of maternal health services, women with a secondary and above education tended to have better antenatal care (89.17 % received prenatal care, 50.69 % had antenatal checks more than four times) and 7.77 % had a cesarean section.

**Table 1:** Basic characteristics of participants^a^

	Level of Education		
	Below Primary and None*N* = 1,873	Primary*N* = 3,408	Secondary and Above*N* = 3,279
Mean age, years (SD)	30.22(0.33)	29.30(0.26)	27.54(0.19)
Urban, %	14.60	22.63	58.56
Wealth Group, %			
Very Poor	39.63	31.64	13.37
Poor	28.97	27.99	14.35
Middle	21.06	24.13	16.04
Rich	10.34	16.24	56.24
Marital Status,%			
Currently Married	88.69	86.68	82.19
Formerly Married	6.24	7.51	5.81
Never Married	5.08	7.64	10.16
Age at first marriage,^b^ %			
≤14	12.74	13.49	8.06
>14, ≤ 16	16.47	21.50	15.39
>16, ≤ 18	20.69	24.43	20.29
> 18	50.10	40.57	56.26
Ever Smoke, %	1.62	1.78	1.59
Ever Use Alcohol, %	24.86	34.10	33.63
Has Disability, %	2.14	3.86	2.54
Has Insurance, %	0.37	1.11	6.93
Maternal Healthcare Utilization, %			
Received Prenatal Care	73.66	79.43	89.17
Antenatal Care ≥ 4	34.56	36.09	50.69
Skilled attendant at birth	76.84	80.43	91.68
Mother received postnatal care	2.81	6.29	3.61
Baby received postnatal care	5.89	7.62	2.38
Cesarean section	3.62	3.45	7.77

^a^The tabled figures here all considered weight by employing “svy” command in Stata

^b^585 participants had never been married in the dataset. Thus, the data were only available for 7,975 participants for the variable “age at first marriage”

**Fig. 1:**
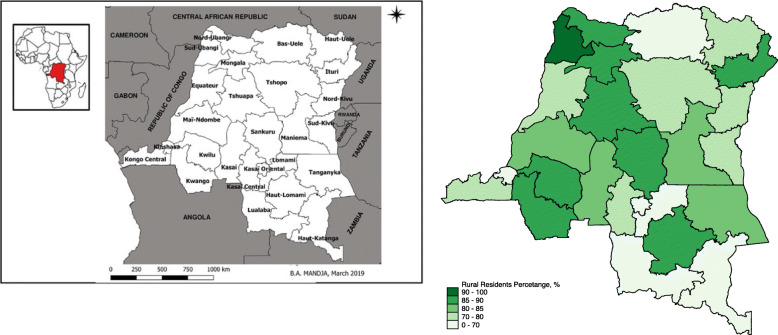
DRC map with 26 administrative regions (left) and percentage (%) of rural residents in each province in the current study (right)

**Fig. 2:**
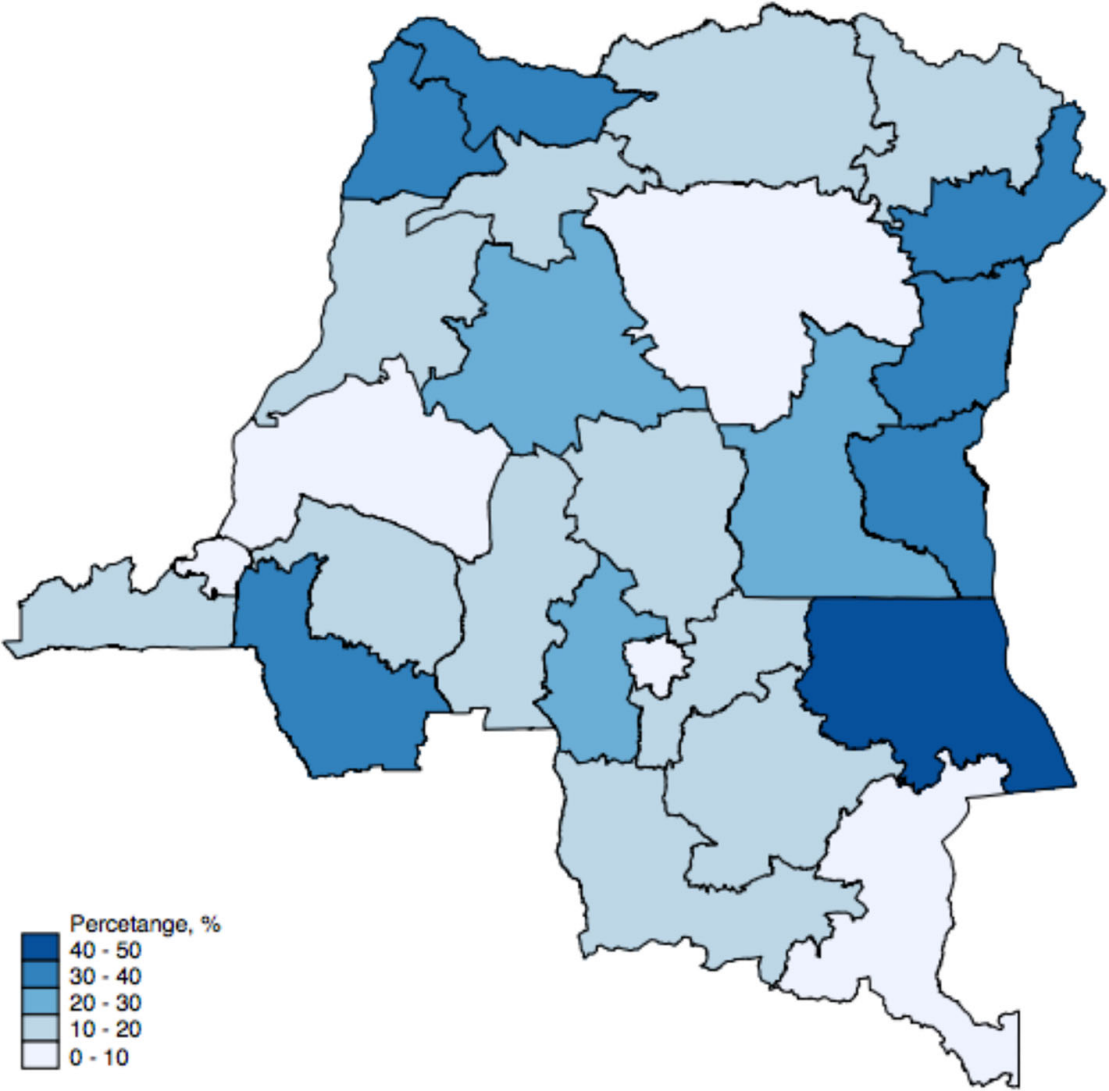
Percentage (%) of women with below primary and no education in each province

**Fig. 3:**
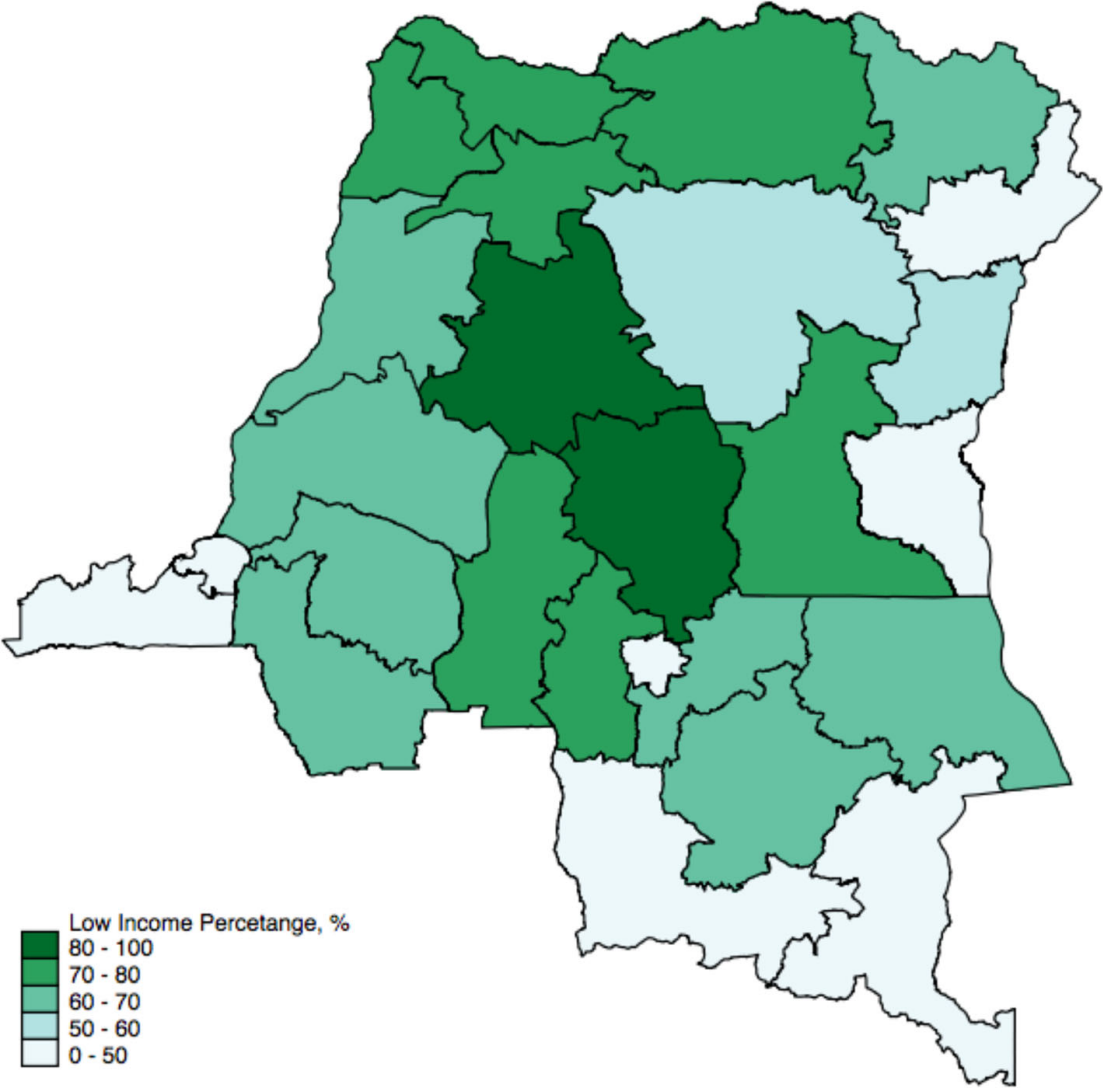
Percentage (%) of residents with low income* in each province. *low income was defined as the bottom two quintiles income

**Fig. 4:**
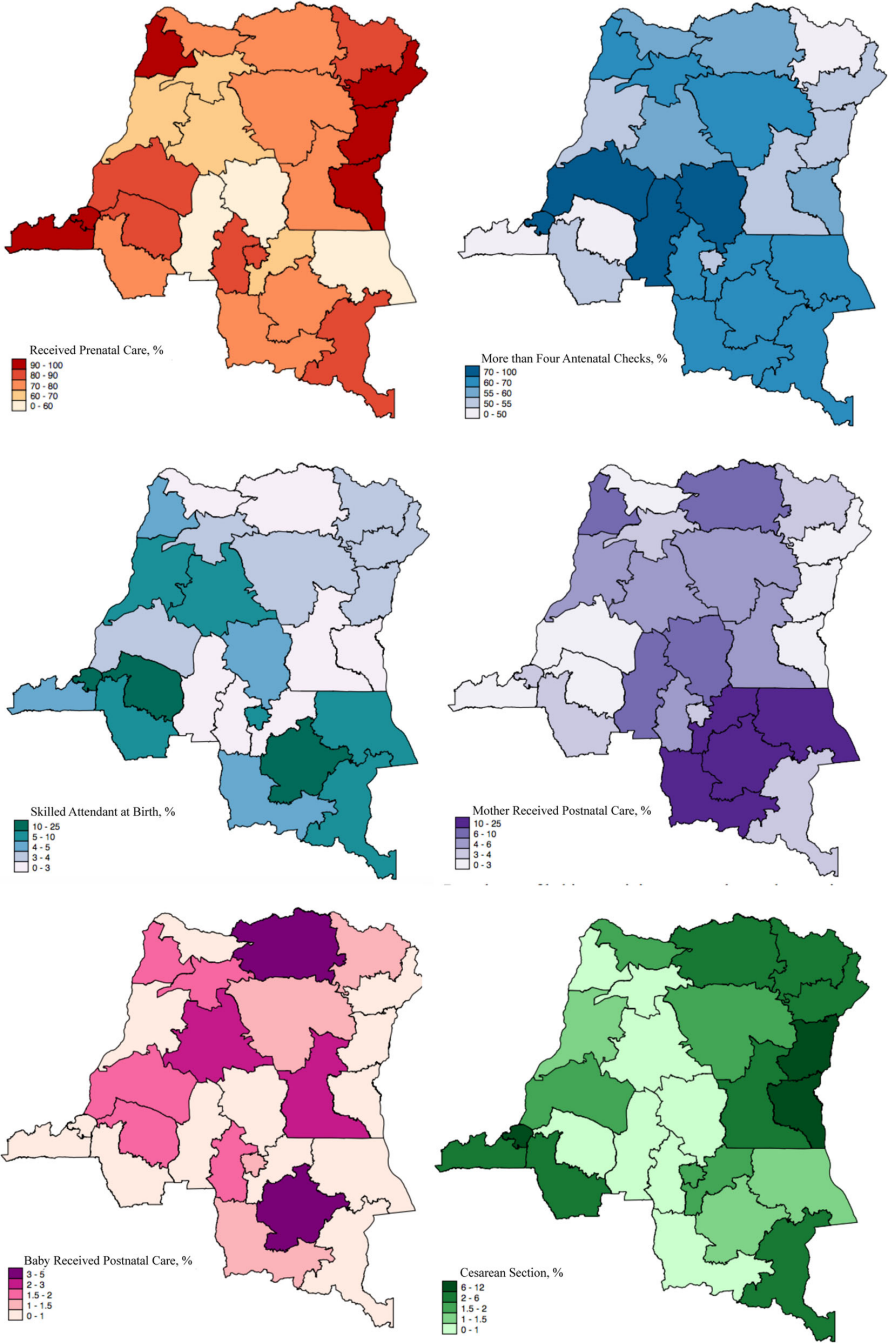
Prevalence of maternal heath indicators in each province

Figures 1 and 2, and 3 present the geographical distribution of the percentage of rural residents, women with below and no education, and residents with low income in each province. The majority of the participants are from rural areas, with most provinces having more than 85 % of their residents living in rural areas. The exception here is the capital, Kinshasa, where all the residents were identified as living in urban areas. The percentage of rural residents is also associated with educational level and wealth status, with more rural areas having less educated and less wealthy populations. Figure 4 presents the geographical distribution of the six indicators for maternal health services utilization. The general pattern is that provinces with less educated, poorer, and rural residents generally have lower access to maternal health services. The detailed geographical distribution of rural residents, education, income level, and maternal health services utilization can be found in Figs. 1, 2, 3 and 4.

### Association between maternal education with maternal health services utilization

Table 2 presents the relationship between maternal health service utilization and maternal education. Overall, a better education was associated with higher utilization of antenatal care. Compared to women in the below primary and no education group, women with a primary school education (OR: 1.38, 95 % CI: 1.04–1.83) and secondary and above education (OR: 2.94, 95 % CI: 2.15–4.03) had higher ORs to received prenatal care. There exists a dose-response relationship whereby the higher the education, the more likely is that women receive prenatal care.

**Table 2:** Relations between educational level and maternal health services utilization^a, c^.

		Unadjusted OR (95 % CI)	
	Primary*N* = 3,408		Secondary and Above*N* = 3,279
Received Prenatal Care	1.38(1.04,1.83)		2.94(2.15,4.03)
Antenatal Care ≥ 4	1.07(0.83,1.38)		1.95(1.52,2.50)
Skilled attendant at birth	1.24(0.96,1.60)		3.32(2.25,4.91)
Mother received postnatal care	2.25(1.09,4.64)		1.90(0.88,4.11)
Baby received postnatal care	2.23(1.24,4.04)		1.05(0.53,2.10)
Cesarean section	0.95(050,1.80)		2.24(1.24,4.06)
	Adjusted OR (95 % CI)^b^		
Received Prenatal Care	1.56(1.18,2.06)		2.49(1.80,3.44)
Antenatal Care ≥ 4	1.08(0.85,1.38)		1.47(1.13,1.91)
Skilled attendant at birth	1.13(0.91,1.41)		1.78(1.29,2.46)
Mother received postnatal care	1.97(0.86,4.55)		2.13(0.85,5.30)
Baby received postnatal care	1.99(1.03,3.85)		1.35(0.60,3.03)
Cesarean section	1.24(0.64,2.40)		1.98(1.07,3.65)

^a^Reference group is the below primary and none education group (*n* = 1,873)

^b^Adjusted for age, urban/rural, province, wealth, marital status, age at first marriage, smoke/alcohol use, and disability

^c^ORs for received prenatal care, ANC ≥ 4, skilled attendant at birth, and cesarean section were calculated with considerations of sampling weight. ORs for mother received PNC and baby received PNC were calculated without weighting due to missing values causing stratum with single sampling unit

Maternal education was positively associated with having skilled attendants at birth. However, the association was only significant when comparing women with secondary and above education to those who had below primary and no education (OR: 1.78, 95 % CI: 1.29–2.46), after adjustment. Compared to women with below primary and no education, women with secondary and above education were more likely to receive cesarean sections (OR: 1.98, 95 % CI: 1.07–3.65). After adjusting for possible confounders, the significant associations between maternal education and maternal health service utilization indicators are observed in whether the mothers had received prenatal care, antenatal care ≥ 4, having skilled attendants at birth, and cesarean section.

### Stratified analysis

To further understand the modifying effects of socioeconomic variables, the analyses were stratified by rural and urban division, low- and middle-high-income, age at first marriage < 18 and ≥ 18. The results were presented in Table 3. The positive association between maternal education and received prenatal care was similar between rural and urban residents and among those who married before and after eighteen years old. It appears that wealth status may also strongly affect the association between maternal education and prenatal care utilization. In the low-income population, the association was significant in both the primary education group (OR: 1.66, 95 % CI: 1.21–2.29) and the secondary and above education group (OR: 2.70, 95 % CI: 1.86–3.93), whereas the association, was only significant in the secondary and above education group (OR: 2.41, 95 % CI: 1.37–4.23) in the middle-high-income population. The association between maternal education and antenatal care ≥ 4 was also only significant in the low-income population. The significant positive association between having skilled attendants at birth and maternal education was only significant in rural areas and low-income populations. The positive association between cesarean section and maternal education level was only significant among rural residents, in the low-income population, and among those whose first marriage was before eighteen years old.

**Table 3:** Relations between educational level and maternal health services utilization, stratified by rural/urban division income, and age at first marriage, adjusted^#^

	Rural*	
	Primary *N* = 3,408	Secondary and Above *N* = 3,279
Received Prenatal Care	1.59(1.16,2.16)	2.35(1.66,3.32)
Antenatal Care ≥ 4	1.19(0.91,1.56)	1.65(1.27,2.16)
Skilled attendant at birth	1.20(0.96,1.51)	1.80(1.26,2.59)
Cesarean section	1.51(0.75,3.03)	2.82(1.62,4.92)
	Urban*	
Received Prenatal Care	1.77(1.02,3.06)	3.17(1.63,6.16)
Antenatal Care ≥ 4	0.87(0.47,1.64)	1.18(0.61,2.28)
Skilled attendant at birth	0.90(0.47,1.74)	1.31(0.70,2.45)
Cesarean section	0.91(0.20,4.19)	1.01(0.25,4.19)
	Low Income (< 40 %)	
Received Prenatal Care	1.66(1.21,2.29)	2.70(1.86,3.93)
Antenatal Care ≥ 4	1.20(0.91,1.58)	1.70(1.21,2.39)
Skilled attendant at birth	1.13(0.88,1.44)	2.05(1.37,3.08)
Cesarean section	2.14(1.05,4.38)	2.04(0.88,4.72)
	Middle-High-Income (> 40 %)	
Received Prenatal Care	1.43(0.82,2.51)	2.41(1.37,4.23)
Antenatal Care ≥ 4	0.94(0.65,1.37)	1.28(0.85,1.94)
Skilled attendant at birth	1.09(0.67,1.77)	1.21(0.73,2.01)
Cesarean section	0.69(0.23,2.10)	1.81(0.67,4.91)
	First Marriage < 18	
Received Prenatal Care	1.61(1.08,2.41)	2.66(1.76,4.02)
Antenatal Care ≥ 4	1.06(0.78,1.45)	1.62(1.18,2.23)
Skilled attendant at birth	1.36(0.99,1.86)	1.92(1.29,2.87)
Cesarean section	1.29(0.51,3.29)	2.60(1.11,6.08)
	First Marriage ≥ 18	
Received Prenatal Care	1.69(1.23,2.33)	2.95(2.04,4.27)
Antenatal Care ≥ 4	1.17(0.82,1.69)	1.64(1.11,2.42)
Skilled attendant at birth	1.11(0.80,1.53)	2.24(1.54,3.27)
Cesarean section	1.28(0.39,4.18)	1.63(0.50,5.32)

^**#**^Reference group is the below primary and none education group (*n* = 1,873)

*Adjusted for age, province, wealth, marital status, age at first marriage, smoke/alcohol use, and disability

** Adjusted for age, urban/rural, province, marital status, age at first marriage, smoke/alcohol use, and disability

*** Adjusted for age, urban/rural, province, marital status, wealth, smoke/alcohol use, and disability

## Discussion

Several important findings are illustrated in the present study. First, from the geographical distribution, we found that the populous capital, Kinshasa, generally has better maternal health services utilization. The combination of a higher percentage of rural residents and the low-income population is associated with a lower maternal healthcare services utilization in the DRC. Maternal education level was positively associated with prenatal care utilization. A higher education level was significantly associated with receiving prenatal care, after being adjusted for potential confounders. The odds ratio for having skilled attendants at birth also increased with maternal education level. The association is similar among rural and urban residents but is stronger among the low-income population. Cesarean section was positively associated with maternal education level. The association is only significant among rural residents and in the low-income population.

The urban populous capital, Kinshasa, where around one-eighth of the population in the DRC is estimated to live, has a better educated and wealthier population, with better maternal health services utilization than the rest of the country. The situation is similar to other sub-Saharan countries where a large number of people gather in the capital city, and the gap in health services between the capital and the rest of the country is large [20, 21]. As shown in the present study, residents in Kinshasa, in general, might have a better socioeconomic situation than the rest of the country, which could be the main reason for better maternal health services utilization. Besides, some other parts of the DRC have been suffering from periodic military conflicts and wars, which worsened the maternal health services utilization and made Kinshasa relatively better-off. For example, a map of conflict made by Verisk Maplecroft (Appendix 1) showed that in 2015, the capital area of Kinshasa was relatively stable whereas other parts of the DRC, especially the eastern part, were affected by different levels of conflicts [22].

Besides, the capital city might have better availability of health facilities, including private health services, and more skilled health personnel compared to other regions. For example, a recent study observed that Kinshasa women have a higher rate of contraceptive use compared to other regions in the country, which might imply that the capital has better public health services [23]. A recent study suggested that even young migrant mothers to the urban areas in sub-Saharan countries utilized maternal healthcare more after their migration [21]. It showed that maternal health services utilization could be improved with a generally better social situation and proper public health policy, even the individual situation remains the same. The DRC has a unique situation in that displacements are common for people to avoid conflicts [24], and people displaced to Kinshasa might have a better healthcare service than in conflict areas. Another study emphasized that the gap within urban areas is also high [25]. The divisions in terms of maternal health services within Kinshasa should be further explored if relevant data become available in future studies. Meanwhile, although the association between maternal health services utilization and prenatal care did not appear to differ in the current study, our results indicated that the maternal health services utilization gap between urban and rural areas, especially between Kinshasa and other parts, do exist. This suggests that public health programs could target Kinshasa and other parts of the DRC separately when designing maternal health interventions. At the same time, good practices that guarantee better maternal health services utilization in Kinshasa should be reviewed and may implement in other parts of the DRC.

A positive association between maternal education level and prenatal care services utilization was observed in the current study, with the association stronger among the low-income population. The overall positive association between maternal education and maternal health services utilization was consistent with previous studies conducted in both developing countries such as India [26] and Ethiopia [27] and developed countries such as Canada [28] and the United States [29]. A recent study conducted in the DRC also indicated that educational level was positively associated with having their first visit to antenatal care facilities within endemic malaria settings [14]. Better maternal education often indicated that women are in an advanced socioeconomic condition, where access to health services is more common. Previous studies also indicated that women with a better education tend to have more active maternal health-seeking behavior, which makes them engage in booking antenatal check appointments, seeking help from professional health services, etc. [12, 14]. Some cultural beliefs may impact the health-seeking behaviors of mothers. For example, a study conducted in Malawi indicated that some African women believe that showing pregnancy at an early stage could harm the fetus as the baby was vulnerable and at risk of being subject to “witchcraft” [30]. They would hide their pregnancy in the early months to avoid being bewitched and thus protect their babies [30]. Such cultural beliefs could be changed by sufficient education and make mothers actively engage in prenatal cares. Additionally, the health literacy level for women with a higher education tends to be better, which indicates that educated women understand the benefits and necessities of prenatal care and have the ability to comprehend recommendations from health authorities [31]. Education level is a unique socioeconomic indicator, in that it is not only a major determinant of socioeconomic status but also have the potential to empower women and increase their health literacy. Thus, long-term public health strategies should reach beyond health and take a comprehensive view, for example, including the enhancement of women’s education.

Maternal education level was positively associated with cesarean section. However, the association was only significant among rural residents and in the low-income population. In many developed countries and emerging economies, countries have a higher than necessary cesarean section prevalence. In these countries, many interventions are trying to reduce the percentage to an ‘essential level’ due to the potential harms of such practices [32]. Meanwhile, low-income countries - especially in Sub-Saharan Africa – seek to raise the percentage, as mothers do not have adequate access to cesarean section as an emergency obstetric intervention [33]. Studies have indicated that a lack of access to or unwillingness to perform cesarean sections is one of the factors for high maternal mortality in low-income countries [33, 34]. Besides, previous research indicated that even when access to cesarean is available, healthcare service providers in sub-Saharan African countries might not have the capacity or the finances to appropriate meet the demand for the procedure, which could lead to surgical infections and cause an increase in mortality [33, 35]. WHO has recommended an optimal cesarean section rate of 10 ~ 15 % as appropriate since 1985 [36], as a below 10 % cesarean section rate was associated with a high maternal mortality rate in low-income countries [37]. In the current study, the cesarean section rate for mothers with secondary and above education is 6.92 % and the rates for the other two education groups are below 3 %, which were all below the optimal level. This indicated that emergency obstetric interventions are not fully accessible in the DRC and interventions should strive to increase the accessibility of cesarean sections, especially for poorly educated populations. Consistent with the present findings, a study conducted in India observed that mothers with higher education had better access to cesarean Sec. [38] and a review in Southern Asia and sub-Saharan Africa indicted that wealth status is an important associate for access to cesarean Sec. [39]. In the current analysis, women with higher education tend to have skilled birth attendants, whose presence are essential for deciding to perform emergency obstetric interventions [40]. The association of maternal education and cesarean section were stronger among rural residents and in the low-income population, which might indicate that in a resource-poor situation, education is especially important in obtaining essential maternal services. Emergency obstetric intervention is an important composite of maternal health services utilization and serves crucial functions in reducing maternal mortality [33]. Nevertheless, current maternal health programs focus more on pre-/post- pregnancy cares rather than providing essential access to necessary emergency obstetric interventions. Future interventions that aim to reduce maternal deaths in the DRC should also consider integrating cesarean section as a part of maternal health programs.

This study is based on a large and population-based survey, which makes it one of the first studies conducted in the DRC to have nationwide maternal health policy implications. It is also one of the first studies conducted in a sub-Saharan African country to focus mainly on the association between maternal education and maternal health services utilization. The results from this study can contribute to the policymaking of both the DRC government and international development partners, as well as contribute to the Sustainable Development Goals (SDGs) in the DRC. Admittedly, there are some potential limitations to the study. First, causal inference between maternal education and maternal health services utilization is limited due to the study being cross-sectional. Further cohort studies are needed in order to establish a causal relationship. Studies using pooled cross-sectional MICS data could also be conducted. Secondly, due to the uniqueness of the DRC, the concept of education could be different, as some participants may receive education inside the community instead of having formal education at school. Illiteracy in the DRC is high even among primary school graduates, which makes the contribution of education to women’s health literacy opaque. Lastly, the survey was based on participants’ recall of their recent birth events, which may create recall biases.

## Conclusion

This study observed that maternal education is positively associated with better maternal health services utilization in terms of access to prenatal care and emergency obstetric care. The strength of the association differs across socioeconomic groups. We also found a geographical disparity in maternal health service utilization between rural and urban areas, including a gap between Kinshasa and other areas, which should be addressed by public health policies. Our results also indicate that the mother’s education level is an important associate for receiving appropriate maternal healthcare. Education is also an important aspect of women’s empowerment programs. Long-term public health strategies should consider women’s educational status to increase access to maternal health services. Future health interventions on maternal healthcare promotion in the DRC should take into consideration the effect of maternal education and develop specific interventions strategies according to the mother’s educational level, among other socioeconomic indicators. For example, relevant programs need to consider how maternal education affect the utilization of maternal healthcare services and then modify and stratify the interventions accordingly. The program intervention according to educational attainment. Besides, multiple MICS surveys had been conducted prior to the 2017/2018 survey used in the current research. Further pooled cross-sectional studies can be conducted to explore the trend in maternal healthcare utilization and how maternal education may affect such a trend.

## Supplementary Information

**Additional file 1:** Appendix 1 Democratic Republic of the Congo: Conflict and political violence (Map of the week, 26/01/2015).* *Made by Maplecroft and copied from https://reliefweb.int/map/democratic-republic-congo/democratic-republic-congo-conflict-and-political-violence-map-week, accessed on May 8th, 2021.

## Data Availability

The datasets used and/or analysed during the current study available from the corresponding author on reasonable request.
