# Supplementary material for: Maternal education level and maternal healthcare utilization in the Democratic Republic of the Congo: an analysis of the multiple indicator cluster survey 2017/18
Source: BMC Health Serv Res. 2021 Aug 21;21:850. doi: 10.1186/s12913-021-06854-x (PMC8380349; doi:10.1186/s12913-021-06854-x)
Supplement: Supplementary file 1 — Additional file 1: Appendix 1 Democratic Republic of the Congo: Conflict and political violence (Map of the week, 26/01/2015).* *Made by Maplecroft and copied from https://reliefweb.int/map/democratic-republic-congo/democratic-republic-congo-conflict-and-political-violence-map-week, accessed on May 8th, 2021. [file 12913_2021_6854_MOESM1_ESM.pdf]

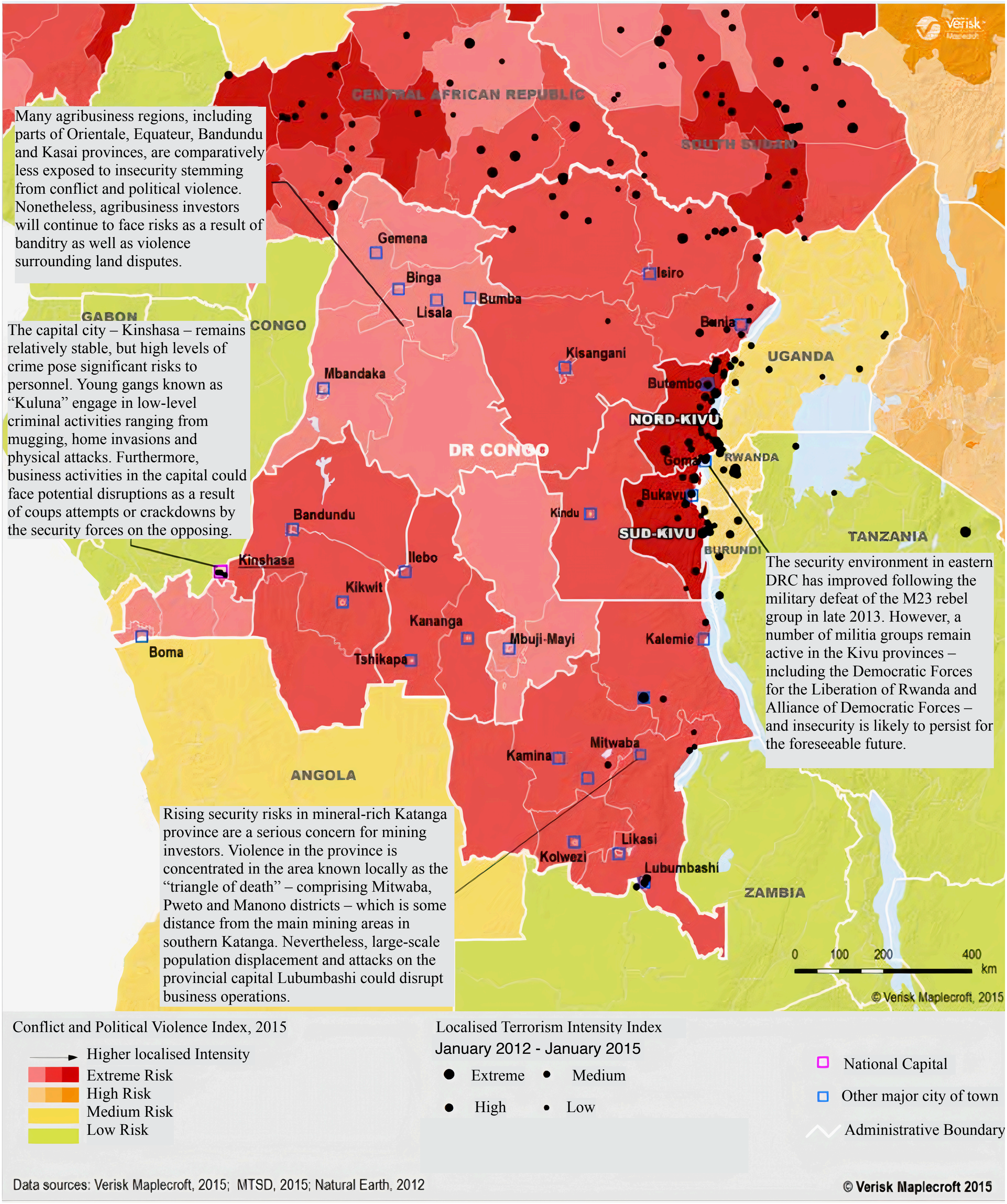
CENTRAL AFRICAN REPUBLIC
SOUTH SUDAN
Many agribusiness regions, including parts of Orientale, Equateur, Bandundu and Kasai provinces, are comparatively less exposed to insecurity stemming from conflict and political violence. Nonetheless, agribusiness investors will continue to face risks as a result of banditry as well as violence surrounding land disputes.
GABON
CONGO
The capital city – Kinshasa – remains relatively stable, but high levels of crime pose significant risks to personnel. Young gangs known as "Kuluna" engage in low-level criminal activities ranging from mugging, home invasions and physical attacks. Furthermore, business activities in the capital could face potential disruptions as a result of coups attempts or crackdowns by the security forces on the opposing.
Gemena
Binga
Lisala
Bumba
Isiro
Bunia
Kisangani
Mbandaka
UGANDA
Butembo
NORD-KIVU
DR CONGO
Goma
RWANDA
Bukavu
Kindu
Bandundu
SUD-KIVU
BURUNDI
TANZANIA
Kinshasa
Ilebo
Kikwit
Kananga
Mbuji-Mayi
Kalemie
Boma
Tshikapa
The security environment in eastern DRC has improved following the military defeat of the M23 rebel group in late 2013. However, a number of militia groups remain active in the Kivu provinces – including the Democratic Forces for the Liberation of Rwanda and Alliance of Democratic Forces – and insecurity is likely to persist for the foreseeable future.
Kamina
Mitwaba
ANGOLA
Rising security risks in mineral-rich Katanga province are a serious concern for mining investors. Violence in the province is concentrated in the area known locally as the "triangle of death" – comprising Mitwaba, Pweto and Manono districts – which is some distance from the main mining areas in southern Katanga. Nevertheless, large-scale population displacement and attacks on the provincial capital Lubumbashi could disrupt business operations.
Likasi
Kolwezi
Lubumbashi
ZAMBIA
0 100 200 400 km
© Verisk Maplecroft, 2015
Conflict and Political Violence Index, 2015
Higher localised Intensity
Extreme Risk
High Risk
Medium Risk
Low Risk
Localised Terrorism Intensity Index
January 2012 - January 2015
Extreme
Medium
High
Low
National Capital
Other major city of town
Administrative Boundary
Data sources: Verisk Maplecroft, 2015; MTSD, 2015; Natural Earth, 2012
© Verisk Maplecroft 2015
